# TREM2 as a Prognostic Biomarker for Osteosarcoma Microenvironment Remodeling

**DOI:** 10.1155/2023/3677789

**Published:** 2023-02-17

**Authors:** Zhi-Long Shen, Zhao-Yu Chen, Yong Ji, Hao Jiang, Zhi-Peng Zhu, Hao Yuan, Bo Li, Wei Xu, Jianru Xiao

**Affiliations:** ^1^Department of Orthopedic Oncology, Changzheng Hospital, Second Military Medical University, 415 Fengyang Road, Shanghai 200003, China; ^2^The Department of Spine Surgery II, Strategic Support Force Medical Center, Beijing, China

## Abstract

The tumor microenvironment (TME) acts as a crucial role in the occurrence and development of osteosarcoma (OS). Despite this, the mechanism controlling the components of immunity and stroma in the tumor microenvironment remains a mystery. To conduct this study, we download and collate transcriptome data from the TARGET database, whose full name is Therapeutically Applicable Research to Generate Effective Treatments, as well as available clinical information of OS. The CIBERSORT and ESTIMATE methodology are used to acquire the proportions of components of immunity and stroma and tumor-infiltrating immune cells (TICs). Protein-protein interaction (PPI) networks and Cox regression analysis are used to select differentially expressed genes (DEGs). A prognostic biomarker is determined by intersecting univariate COX and PPI results, which lead to the finding of Triggering receptor expressed on myeloid cells-2 (TREM2). Based on the next analysis, TREM2 expression is positively correlated with OS survival time. Immune function-related genes have enrichment in the group with high expression of TREM2, according to gene set enrichment analysis (GSEA). The percentage of TICs by CIBERSORT methodology revealed that the expression of TREM2 is positively associated with follicular helper T cells, CD8-positive T cells, and M2 macrophages and negatively correlated with plasma cells, M0 macrophages, and naive CD4-positive T cells. All results suggest a possible integral role of TREM2 in the immune-related events of TME. Therefore, TREM2 may be a potential indicator of remodeling of TME in osteosarcoma, which is useful and helpful in predicting the clinical prognostic outcome of OS patients and provide a unique perspective for immunotherapy for OS.

## 1. Introduction

Chiefly affecting children and young adults and occupying about nine percent of cancer-related deaths in youngsters whose age range is between 10 and 24 years old [1, 2], the exact cause of osteosarcoma is still unclear as a primary malignant bone tumor. In addition, the local invasiveness and metastasis of osteosarcoma remain an enormous challenge of therapy and poor prognosis [3]. With the advent of adjuvant and neoadjuvant chemotherapy, the five-year survival rate of OS had a substantial increase which is upto about 70% since the 1970s, but after lung metastasis, the five-year survival rate still maintains as low as 20–30% [4]. In addition to this, osteosarcoma is highly heterogeneous which makes the prediction of treatment outcomes complicated [5]. The OS includes distinct histological subtypes: osteoblastic, chondroblastic, fibroblastic, giant-cell rich, epithelioid, small-cell, and telangiectatic types [4]. Therefore, exploring new diagnostic and predictive biomarkers and validating more therapeutic targets are continuously essential and critical.

Acting decisive roles in tumor occurrence, progression, metastasis, and sensitivity to therapy, TME has aroused tremendous interest in basic and clinical research as a therapeutic target in cancer [6]. Resident stromal cells and recruited immune cells are the primary component of TME in OS. There is convincing evidence to prove that the stromal cell acts a prominent role in angiogenesis and the remodeling of extracellular matrix in tumors [7]. The occurrence, growth, and progression of tumors are critically affected by the mutual effects between host tumor cells and stromal cells. However, the stromal components of different tumors vary widely. The understanding of the mechanism of crosstalk among tumors is still at a low level [7]. In the meantime, several studies keep close tabs on how immune-related cells impact tumor occurrence, growth, and progression. An increasing number of research studies reveal that TICs acted as an up-and-coming indicator for the understanding and therapeutic effects of TME [8]. Studies have shown that osteosarcoma's immune environment is primarily composed of T-lymphocytes and macrophages. Osteosarcoma cells can control the recruitment, differentiation, and development of immune-infiltrating cells, which results in a local environment of immune tolerance. This kind of environment is favorable to the development of tumors, the resistance of drugs, and even metastases [9, 10]. Therefore, to properly demonstrate the mechanism of TME immune and stromal components regulation, precise genetic analysis is a research hotspot as well as a challenge.

In our study, CIBERSORT and ESTIMATE methodology is used to count on the proportions and composition of the components of immunity and stroma of OS patients from the TARGET database and selected interesting biomarker TREM2. Several researchers identified the TREM2 receptor as a dominating signaling hub of pathology-induced immunity, which can sense tissue damage and activate robust remodeling immunity as responding to it [11]. By playing a part in tumor-associated macrophages (TAMs) and myeloid-derived suppressor cells (MDSCs), TREM2 participated in facilitating an immune-suppressive TME in numerous cancers, including lung cancer, gastric cancer, and glioma [11–13]. In our study, embarking on a comparison between components of immunity and stroma in TME, differentially expressed genes (DEGs) are generated, which revealed that TREM2 may be a potential biomarker of TME remodeling in osteosarcoma.

## 2. Data Collection and Processing

### 2.1. Data Source

All data of transcriptome RNA-seq of 101 OS samples and clinical data (including age and sex) of 253 clinical cases are downloaded and collated from the TARGET database (https://ocg.cancer.gov/programs/target) on May 24, 2022. In the genetic screening phase, we used all transcriptomic data, but in the prognostic analysis, we used only those data that had both transcriptomic data and survival status (survival status and survival time). After integration, a total of 95 samples had both transcriptome and survival data. There were 55 men and 40 women. Eleven patients were younger than 10 years, 62 were between 10 and 18 years, and 22 were older than 18 years.

### 2.2. Calculation of Three Kinds of Score

To estimate the components of immunity and stroma in TME for every sample, the ESTIMATE algorithm is loaded with estimate package [14] in R software (version 4.2.0). The three kinds of scores (ImmuneScore, StromalScore, and ESTIMATEScore) increase with the increase of each of the three levels (immunity-related, stroma-related, and the summation of both), respectively. The larger the scores are, the higher the respective composition of the corresponding TME components is.

### 2.3. Survival Analysis

We combined the three kinds of scores in TME with survival information of OS patients, using the Limma package in R. On account of the median value immune score and stromal score, ninety-five OS patients are split into two different groups, low- and high-score groups, respectively. Using the survival and survminer packages in R software, survival and survminer analyses are calculated. Survival curves are plotted using the Kaplan–Meier methodology, and statistical significance is ascertained by log-rank test; *P* < 0.05 is accepted as significant statistically.

### 2.4. Identification of Differently Expression Genes between the Low and High Groups

The median value allows the sample to be divided into two equal parts, so we use it as the split line. One hundred and one patients are distinguished as low or high scores, respectively, in comparison with the median ImmuneScore and StromalScore values. Differences between high- and low-scoring samples are achieved by using the R and limma package, also low and high subgroups are compared to obtain the corresponding differentially expressed genes. Genes with FDR <0.05 and log_2_-transformed fold change >1 (high subgroup/low subgroup) are regarded as significantly differentially expressed genes.

### 2.5. Enrichment Analyses of GO and KEGG

The enrichplot, clusterProfiler, and ggplot2 packages of R software are used to classify 118 DEGs according to genomic annotation information, i.e., gene ontology (GO) and Kyoto encyclopedia of genes and genomes (KEGG). Genomic annotation information with both *p* and *q* values < 0.05 are regarded as an important and statistically significant role in the development and progression of osteosarcoma.

### 2.6. Heatmaps

R with the heatmap package is applied to establish the heatmap of DEGs.

### 2.7. The Difference Analysis of Scores with Clinical Characteristics

Data on clinical information of OS patients are also of interest. R software is used to perform statistical analysis, and Wilcoxon or Kruskal–Wallis rank sum tests are used to determine whether there are statistical differences between clinical indicators between two groups.

### 2.8. Establishment of a PPI Network

PPI networks reveal the interactions between proteins, and we chose to use the STRING database to construct the corresponding network graphs. What is worth mentioning is that the nodes used to set up the network contain only those nodes whose confidence level of interaction is greater than 0.9.

### 2.9. Analysis of COX Regression

Univariate Cox regression analysis allows initial screening out of potentially nonsignificant variables, which is achieved through R software and survival package. As shown, those ascertained and significant genes met *p*  <  0.05 in both analyses of univariate Cox and Kaplan–Meier tests.

### 2.10. Gene Set Enrichment Analysis

Briefly, GSEA can determine the contribution of a predefined gene set to the phenotype, our gene set is all transcriptomic data as described previously, and the analysis is based on the C7 and HALLMARK target sets (v6.2). Just gene sets with corrected *p*  <  0.05 and FDR *q* < 0.05 are regarded as significant sets. All GSEA analyses are performed on GSEA-4.2.3 software.

### 2.11. TIC Profile

The TIC abundance profiles of tumor samples can reflect the immune cell composition in osteosarcoma to some extent and can be calculated by CIBERSORT. The calculated results are screened, and only samples of *p* < 0.05 are retained for subsequent analysis.

### 2.12. Statistical Analysis

All statistical analyses were conducted by R software (version 4.1.3). The Wilcoxon test was used to compare the differences between the two groups. *p* value <0.05 was considered statistically significant.

## 3. Result

### 3.1. Analysis Process of This Study

**Figure 1:**
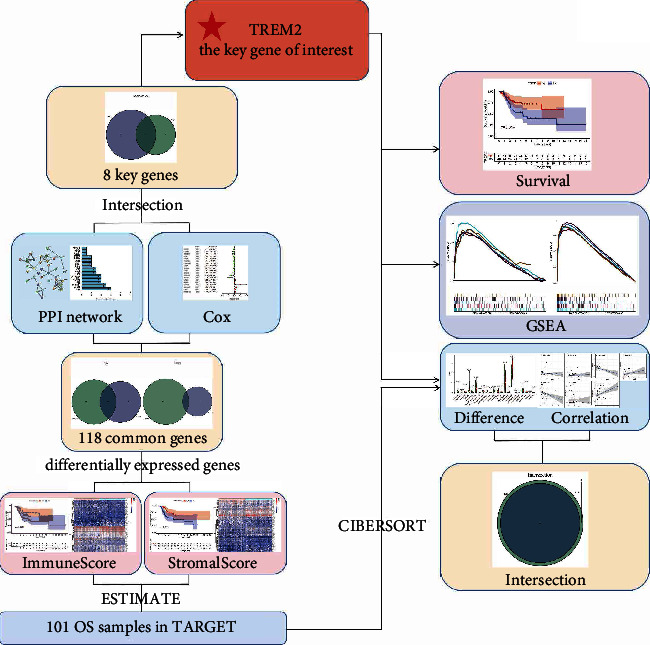
Schematic diagram of the study design.

This study can be divided into two major steps: the discovery of TREM2 and the follow-up study of TREM2 (Figure 1). First, osteosarcoma tissue consists of tumor cells and stromal cells, which correspond to ImmuneScore and StromalScore. Each score is used to divide samples into two groups, respectively, using the median value as the cut-off value, and the intersection of DEGs between the high and low groups of each score is used for subsequent PPI and regression analysis, while the intersection of PPI and regression analysis results in turn, eight key genes (ITGAM, HLA-DMA, LY96, C1QA, C1QB, C1QC, TREM2, and C3AR1) are identified. TREM2 is used as our gene of interest for subsequent studies including survival analysis, GSEA, and analysis of immune-related functions.

### 3.2. Scores Are Associated with OS Patient Survival and Clinical Characteristics

**Figure 2:**
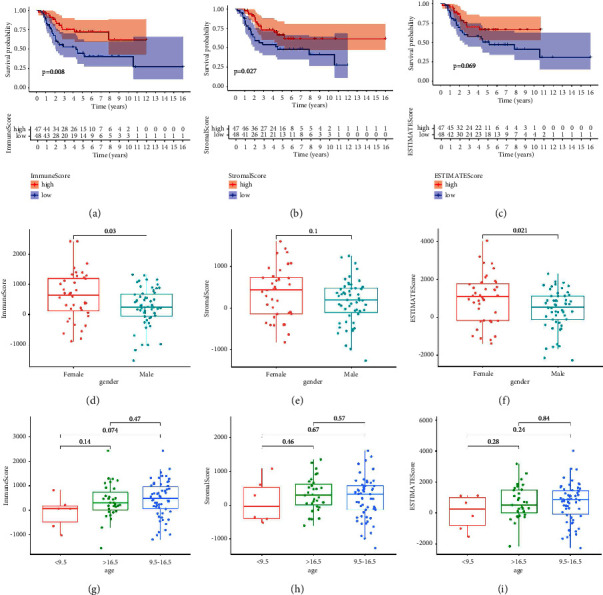
Correlation of scores with the survival and clinical characteristics of patients with osteosarcoma (OS). (a–c) Kaplan–Meier survival analysis for OS patients grouped into high or low score in ImmuneScore, StromalScore, and ESTIMATEScore determined by the comparison with the median, respectively. *p*=0.008, 0.027, 0.069 by log-rank test, respectively. (d–f) Distribution of ImmuneScore, StromalScore, and ESTIMATEScore in gender. The *p*=0.03, 0.1, and 0.021, respectively, by Kruskal–Wallis rank sum test. (g–i) Distribution of ImmuneScore, StromalScore, and ESTIMATEScore in age. The *p* value was calculated by Kruskal–Wallis rank sum test as shown in this figure.

An important indication of whether the immune and stromal ratios are significant in patients with osteosarcoma is the relationship with survival, so we performed a Kaplan–Meier analysis of three kinds of scores, and not surprisingly, the scores correlated positively with survival (Figures 2(a) and 2(b)). To assess the combined composition of two components in TME, we add ImmuneScore and StromalScore to get ESTIMATEScore (Supplement Table 1). Despite the result showing there is no significant correlation between ESTIMATEScore and the overall survival rate (Figure 2(c)), its *p* value is still less than 0.1. These entire results implied that the composition of TME is clinically important and the compositional aspects of TME can forecast patients' prognosis of OS, especially immune and stromal components.

In addition to the survival rate, it is worth discussing whether these three kinds of scores are correlated with other clinical indicators such as age and gender. The results indicated that gender is significantly correlated with ImmuneScore and ESTIMATEScore in patients (*P* < 0.05, Figures 2(d) and 2(f)), except StromalScore (*p*=0.1, Figures 2(e)), while age is not significantly correlated with any score (*p*  >  0.05, Figures 2(g)–2(i)). We found that scores in female patients are higher than in male patients.

### 3.3. Immune-Related Genes Are Mainly Shared DEGs between the ImmuneScore and StromalScore

**Figure 3:**
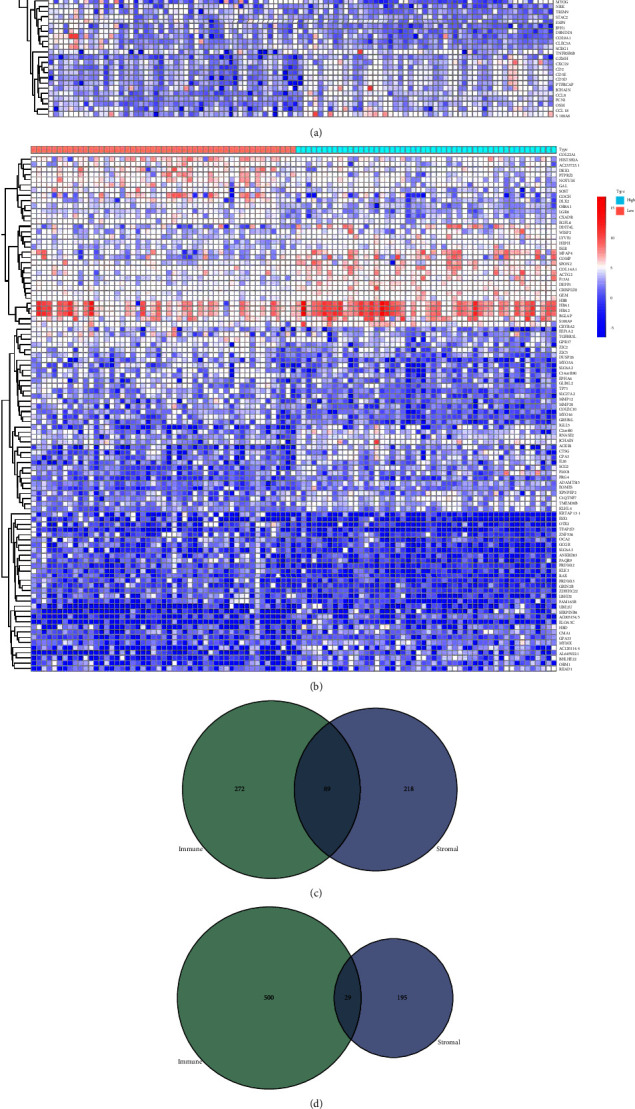
Heatmaps, Venn plots, and enrichment analysis of GO and KEGG for DEGs. (a, b) Heatmap for DEGs generated by comparison of the high-score group vs. the low-score group in ImmuneScore and StromalScore, respectively. Row name of heatmap is the gene name, and column name is the ID of samples which not shown in plot. Differentially expressed genes were determined by Wilcoxon rank sum test with *q* = 0.05 and fold change >1. (c, d) Venn plots showing common upregulated and downregulated DEGs shared by ImmuneScore and StromalScore, and *q* < 0.05 and fold change >1 as the DEGs significance filtering threshold. (e, f) GO and KEGG enrichment analysis for 118 DEGs, terms with p and *q* < 0.05 were considered to be enriched significantly.

Analysis of comparing patients between low and high scores is executed to ascertain if there are definitive genetic profile alterations of components of immunity and stroma in TME. Eight hundred and ninety DEGs (Five hundred and twenty-nine downregulated and three hundred and sixty-one upregulated genes) are received by comparing two groups (low- and high-ImmuneScore patients), with the median value as the cut-off (Figures 3(a), 3(c), 3(d), Supplement Table 2). Correspondingly, five hundred and thirty-one DEGs (Two hundred and twenty-four downregulated and three hundred and seven upregulated) are received from the StromalScore (Figures 3(b)–3(d), Supplement Table 3). Furthermore, twenty-nine low-score downregulated genes and eighty-nine high-score upregulated genes are cross-linked between the ImmuneScore and StromalScore by an analysis of Venn diagrams (Figures 3(c) and 3(d), Supplement Table 4). The entire DEGs (118 genes in all) are deemed as determinants of status in TME. GO enrichment analysis results give evidence that the DEGs ordinarily have a corresponding in terms linked to immunity, including innate and acquired immunity (Figure 3(e), Supplement Figure 1A and 1B). The KEGG enrichment analysis similarly gives evidence of that DEG enrichment in the disease spectrum is related to the immune system, including infection and autoimmune disease (Figure 3(f), Supplement Figure 1C and 1D). Hence, the overall function of differentially expressed genes appears to have a corresponding immune-related event, hinting the participation of immunity-related elements is a principal signature in the TME of OS.

### 3.4. Cross-Tabulation Analysis between Univariate Cox Regression and PPI Network

**Figure 4:**
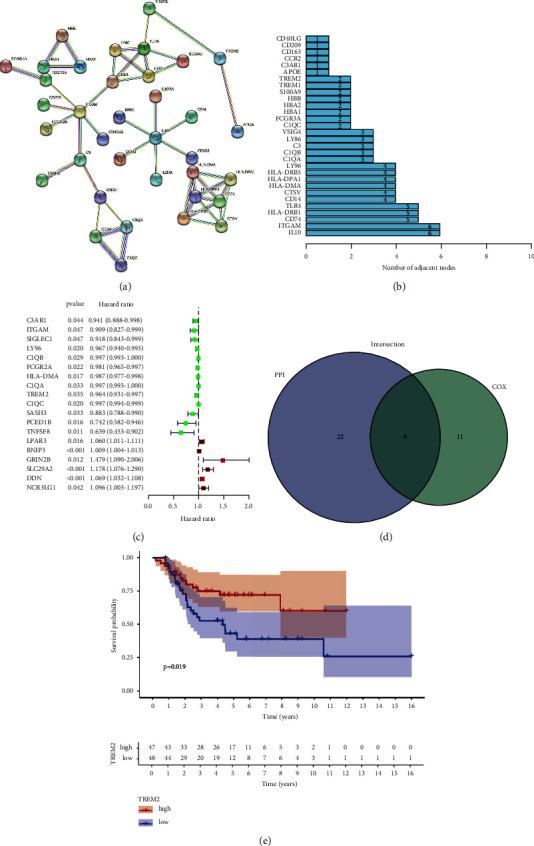
Protein-protein interaction network and univariate Cox analysis. (a) Interaction network constructed with the nodes with interaction confidence value >0.90. (b) The top 30 genes ordered by the number of nodes. (c) Univariate Cox regression analysis with 118 DEGs, listing the top significant factors with *p*  <  0.05. (d) Venn plot showing the common factors shared by leading 30 nodes in PPI and top significant factors in univariate Cox. (e) Survival analysis for OS patients with different TREM2 expression. *p*=0.019 by log-rank test.

To move forward a single step in exploring the latent mechanism, we worked with Cytoscape software to set up the PPI network in the STRING database.  Figure 4(a) show the mutual interplay among the 118 genes, and ranked in the top thirty genes are listed in the picture as rank order (Figure 4(b)). The vital factors impacting the survival of OS patients among 118 DEGs are selected by applying univariate COX regression analysis (Figure 4(c)). Then, these intersecting sets between the core nodes of PPI and the top nineteen Cox regressors is carried out, and eight superimposed factors are in place, which are identified (ITGAM, HLA-DMA, LY96, C1QA, C1QB, C1QC, TREM2, and C3AR1, Figure 4(d)).

### 3.5. Relationships between TREM2 and Survival Time and Clinical Characteristics in OS Patients

Based on previous report, we chose TREM2 for further study [15]. According to the median expression of TREM2 gene, we separated the OS patients into two groups, low- and high-expression TREM2 expression groups. There is a significant difference of survival rate statistically between two groups by the high TREM2 expression group has a higher survival rate than patients with corresponding low expression (Figure 4(e)). What's more, there is no statistical difference between TREM2 expression and clinical characteristics (Supplement Figure 2).

### 3.6. TREM2 as a Potential Indicator of TME Remodeling

**Figure 5:**
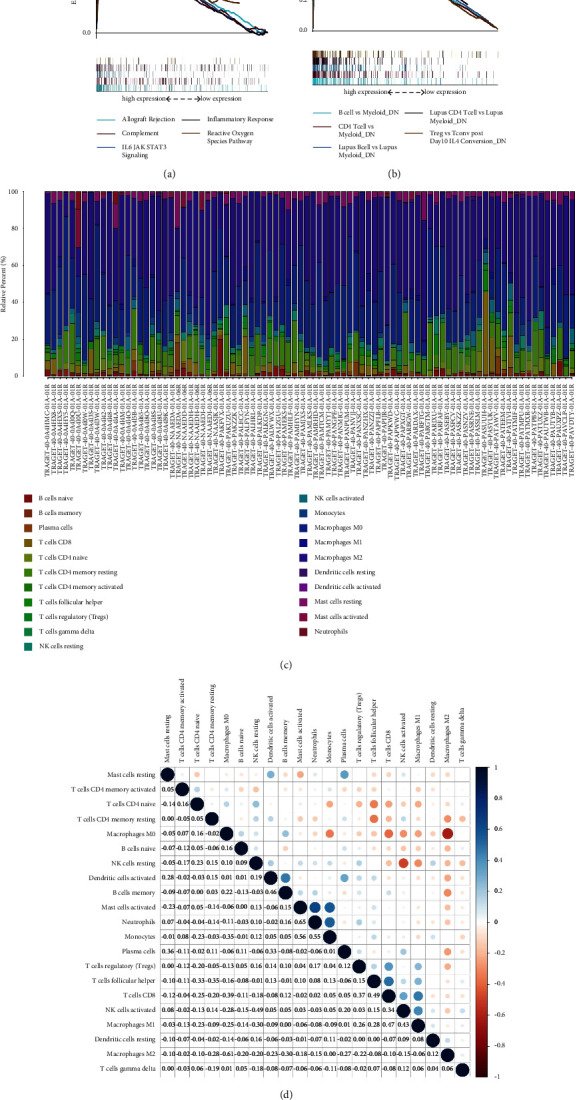
GSEA for samples with high TREM2 expression and low expression and TIC profile and correlation analysis in tumor samples. (a, b) The enriched gene sets in Hallmark and C7 sets by the high TREM2 expression sample. (c) Bar plot showing the proportion of 21 kinds of TICs in OS tumor samples. (d) Heatmap showing the correlation between 21 kinds of TICs and numeric in each tiny box indicating the *p* value of correlation between two kinds of cells. The shade of each tiny color box represented corresponding correlation value between two cells, and Pearson coefficient was used for significance test.

Taking the fact that the levels of TREM2 expression have positive correlation with OS patient survival into consideration, these two groups are in comparison in GSEA. Hallmark and C7 sets of both demonstrated that the groups with high expression of TREM2 have observably more enrichment in immunity-related gene sets, suggesting immunity-related functions, such as the complement response, allograft rejection, IL6/JAK/STAT3 signaling, and acquired immunity are substantially more vibrant (Figures 5(a) and 5(b)). Therefore, it is implicit that the status of the TME can be mirrored by the TREM2 expression.

### 3.7. Correlation Analysis of the Levels of TREM2 Expression and TICs

**Figure 6:**
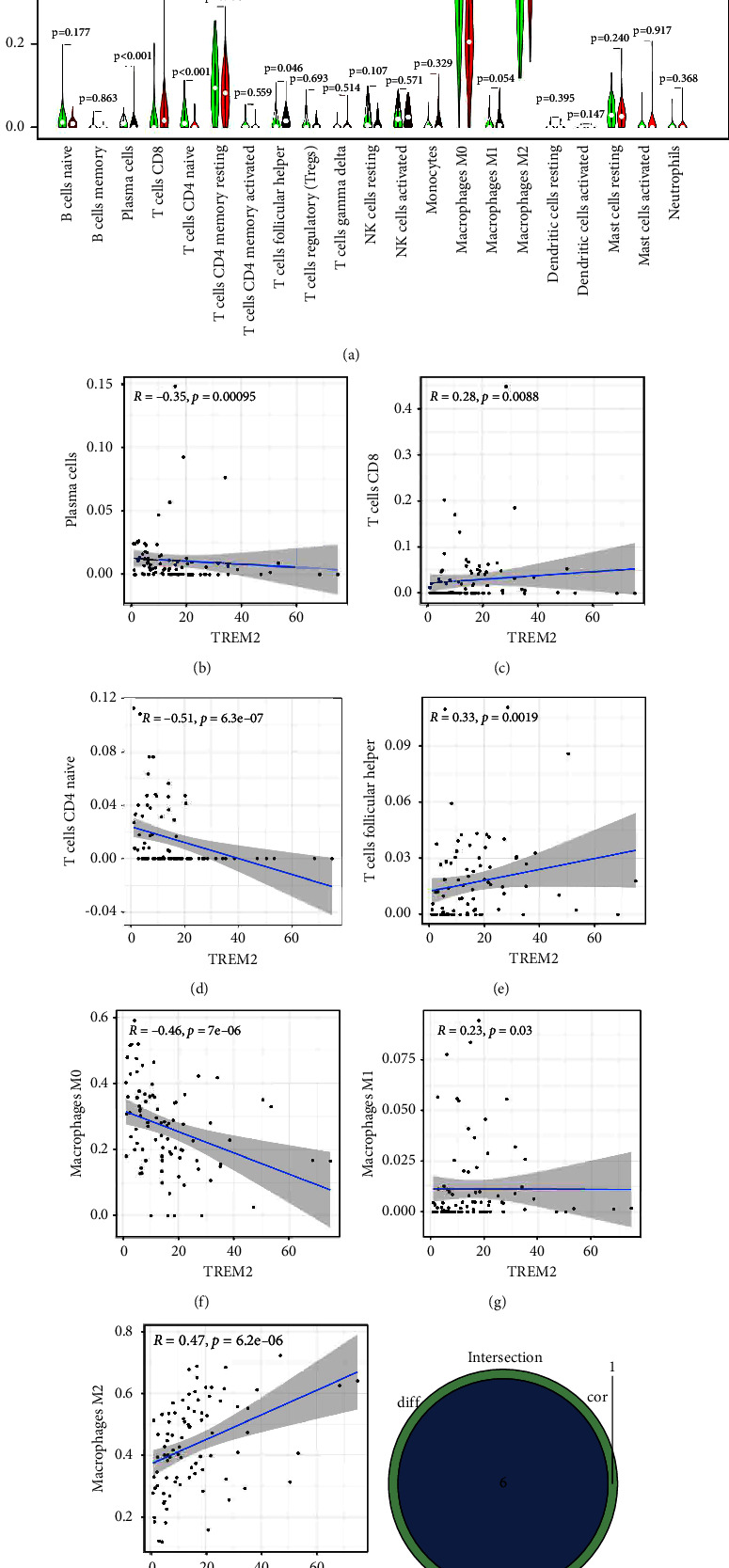
Correlation of TICs proportion with TREM2 expression. (a) Violin plot showed the ratio differentiation of 21 kinds of immune cells between OS tumor samples with low or high TREM2 expression relative to the median of TREM2 expression level, and Wilcoxon rank sum was used for the significance test. (b–h) Scatter plot showed the correlation of 7 kinds of TICs proportion with the TREM2 expression (*p*  <  0.05). The red line in each plot was fitted linear model indicating the proportion tropism of the immune cell along with TREM2 expression, and Pearson coefficient was used for the correlation test. (i) Venn plot displayed eight kinds of TICs correlated with TREM2 expression codetermined by difference and correlation tests displayed in violin and scatter plots, respectively.

To move forward a single step in confirming the relevance between TME and expression levels of TREM2. CIBERSORT methodology is utilized to acquire the immune subpopulation composition of tumor-infiltrating. The establishment of twenty-two kinds of immunity-related cell profiles is executed as follows (Figure 5(c)), and the relevance among TICs is figured up (Figure 5(d)). The discrepancy and connection between the expression of TREM2 expression and the proportions of TICs are analyzed. Six kinds of TREM2-related TICs are obtained (Figures 6(a)–6(h)). Of these, three types of TICs are associated positively with the expression of TREM2, including CD8-positive T cells, follicular helper T cells, and M2 macrophages. Three types of TICs, including plasma cells, naive CD4-positive T cells, and M0 macrophages, are associated negatively with the expression of TREM2. What's more, there are 6 kinds of TICs of TREM2 expression (Figure 6(i)). These findings are a further indication of the effect of TREM2 expression levels on TME immunoactivities.

## 4. Discussion

In the current study, genes of the tumor microenvironment that related to the survival of OS patients from the TARGET database are what we attempted to appraise. TREM2 is appraised to be engaged in immunity-related activities. More significantly, a battery of research on bioinformatics revealed that TREM2 is a prognostic biomarker for osteosarcoma microenvironment remodeling.

The tumor microenvironment played a pivotal part in tumorigenesis and its progression. It is of strategic meaning to detect the underlying therapeutic targets which can contribute to the remodeling and facilitating the transition of the tumor microenvironment from a developmental state to an inhibitory state.

Numerous research studies had elucidated the significance of tumor microenvironment in tumorigenesis [16]. In osteosarcoma tumor microenvironment-related literature, we take notice that the connection between the score of immunity and survival state has been investigated, and C3AR1, PPARG, PDK1, IGHG3, and C1Q are recognized as prognostic biomarkers [17–19]. The immune components in TME sever the purpose of the prognosis of patients by analyzing the OS data in the TARGET database. In particular, the composition of immunity and stroma in TME has a strong correlation with the overall survival in OS patients. These results demonstrated and emphasized the importance of pursuing the connection between stromal cells and tumor cells, which will give a novel perception for discovering and developing more efficient therapy. What's more, this paper also substantiated that TICs have relevance with the clinical prognostic outcome of OS [20]. The relevance offers a brand new theoretical footstone for the evolution of more efficient immunotherapeutic methods.

For the last few years, tremendous progress was acquired in immunotherapy, and the inhibition of immune checkpoint inhibitors (ICIs) in OS made significant progress [21, 22]. However, the inhibition of immune checkpoint inhibitor (ICI) immunotherapy for osteosarcoma (OS) is severely restricted by the lacking of immunogenicity and poor T cell infiltration [23, 24]. Therefore, the immunotherapy of OS is in urgent need of some novel candidate exploitation. Here, the decreased expression of TREM2 has a significant association with poor prognosis by analyzing the transcriptomic of OS in the TARGET database. Consequently, we will center on the relevance between the expression of TREM2 and TME to supply a novel treatment idea for OS immunotherapeutic methods.

As a dominating signaling hub of pathology-induced immunity, TREM2 catch the attention of the leading role of myeloid cells in various pathological processes which can mediate immunosuppression [25]. Many markers of tissue injury are ligands for the TREM2 receptor, and binding of the TREM2 receptor and ligand contributes to cell survival and resistance to inflammation, affecting cell phenotype by regulating phagocytosis and metabolism [15]. In cancer research, TREM2 is observed in macrophages beyond 200 cancer cases of humans in fostering an immune-suppressive TME [15]. There, TREM2 is perhaps a biomarker to alter tumor bone marrow infiltration and reinforce immunotherapy of ICIs [25].

CIBERSORT methodology is applied to accomplish the analysis of the proportion of TICs and completed the composition of twenty-two profiles of immune cells. The results exhibited that macrophages accounted for the highest proportion in the TME of OS, especially M2 macrophages. The fraction of M2 macrophages in high expression groups of TREM2 is higher, which may have a relation to the immune-suppressive TME. In addition, CD8-positive T cells and follicular helper T cells have a positive correlation with the differential expression of TREM2. Plasma cells, naive CD4-positive T cells, and M0 macrophages have a negative correlation with the expression of TREM2. All results suggest that the differential expression of TREM2 is linked to the levels of immune cell infiltration and is a critical target for ameliorating the prognosis of OS. As an attractive biomarker for modulation of individual immunotherapy who are intractable to therapy of ICIs and have a TME rich in TAM, TREM2 is tightly associated with TAMs [26].

Applying the ESTIMATE algorithm, functional enrichment analysis is applied to acquire the gene of the tumor microenvironment of OS in the TARGET database. TREM2 catches our eye as a potential prognostic biomarker for OS patients. What is of interest is that, although TREM2 may mediate the immunosuppressive tumor microenvironment through macrophage M2 polarization, its expression level has positive relevance with the overall survival time of osteosarcoma patients. Further research is indispensable to disclose the mechanism of regulating and exploit novel immunotherapeutic strategies.

## Data Availability

The datasets analyzed for this study can be found in the TARGET database (https://ocg.cancer.gov/programs/target).

## Supplementary Materials

Supplementary Materials The Supplementary Figures and Tables for this article can be found online at: https://doi.org/10.6084/m9.figshare.21972614.v1. Supplementary Figure 1: the GO and KEGG enrichment analysis. (A, B) The GO enrichment analysis. (C, D) The KEGG enrichment analysis. Supplementary Figure 2: relationship between TREM2 expression and clinical features. (A) Relationship between TREM2 expression and age. (B) Relationship between TREM2 expression and gender. Supplementary Table 1: immune infiltration score. The row is the ID of the sample and the column is the score, including StromalScore, ImmuneScore, and ESTIMATEScore. Supplementary Table 2: differentially expressed genes between high and low ImmuneScore groups. The row is the gene name, and the column is the fold change after taking the logarithm with the base of 2 and the adjusted *P* value in turn. Supplementary Table 3: differentially expressed genes between high and low StromalScore groups. The row is the gene name, and the column is the fold change after taking the logarithm with the base of 2 and the adjusted *P* value in turn. Supplementary Table 4: common differentially expressed genes shared by ImmuneScore groups and StromalScore groups. The row is the gene name and the column is the fold change after taking the logarithm with the base of 2.
