# Supplementary figures and images for: TREM2 as a Prognostic Biomarker for Osteosarcoma Microenvironment Remodeling
Source: J Oncol. 2023 Feb 17;2023:3677789. doi: 10.1155/2023/3677789 (PMC9957636; doi:10.1155/2023/3677789)

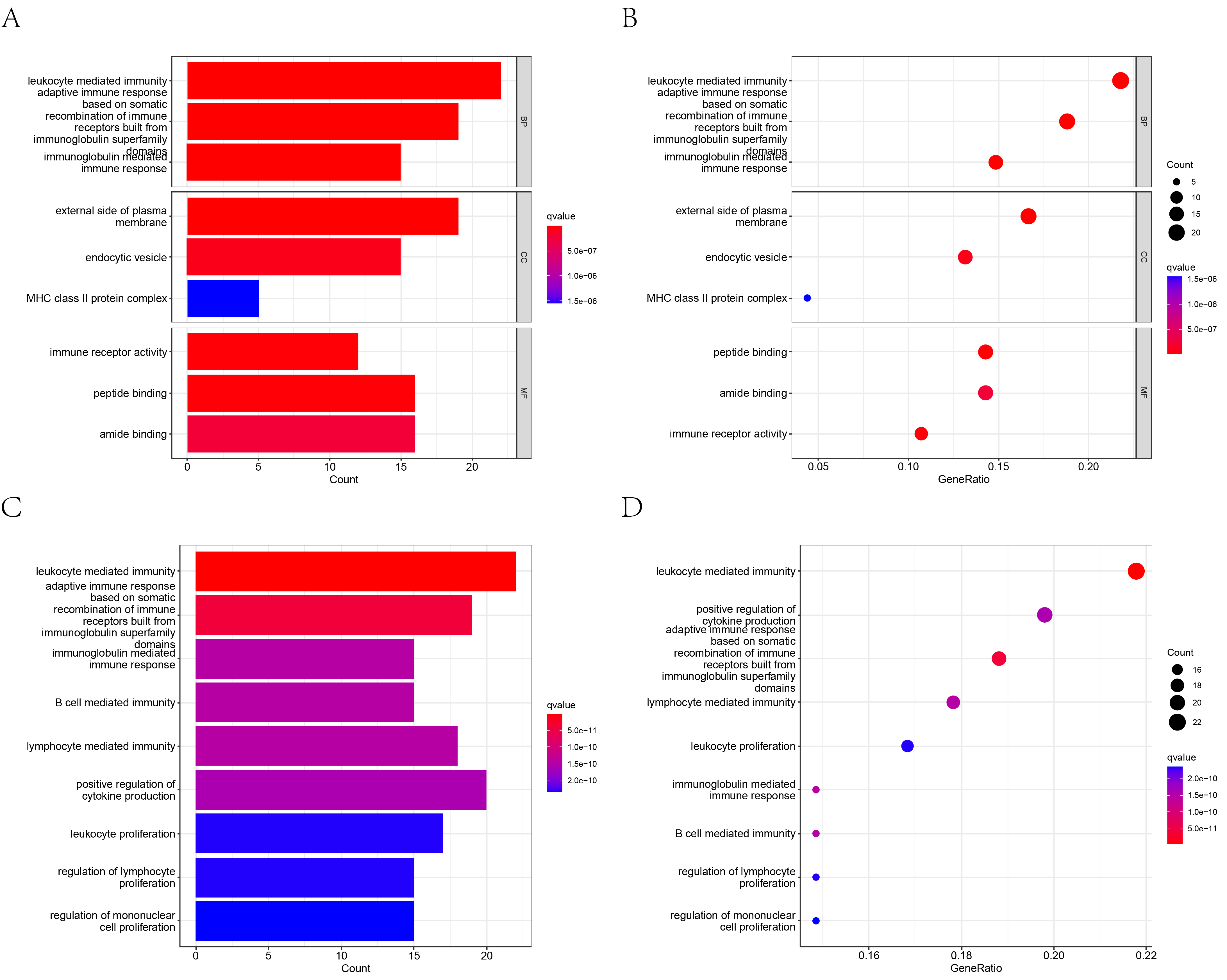

Supplement: Supplementary Materials — The Supplementary Figures and Tables for this article can be found online at: https://doi.org/10.6084/m9.figshare.21972614.v1. Supplementary Figure 1: the GO and KEGG enrichment analysis. (A, B) The GO enrichment analysis. (C, D) The KEGG enrichment analysis. Supplementary Figure 2: relationship between TREM2 expression and clinical features. (A) Relationship between TREM2 expression and age. (B) Relationship between TREM2 expression and gender. Supplementary Table 1: immune infiltration score. The row is the ID of the sample and the column is the score, including StromalScore, ImmuneScore, and ESTIMATEScore. Supplementary Table 2: differentially expressed genes between high and low ImmuneScore groups. The row is the gene name, and the column is the fold change after taking the logarithm with the base of 2 and the adjusted P value in turn. Supplementary Table 3: differentially expressed genes between high and low StromalScore groups. The row is the gene name, and the column is the fold change after taking the logarithm with the base of 2 and the adjusted P value in turn. Supplementary Table 4: common differentially expressed genes shared by ImmuneScore groups and StromalScore groups. The row is the gene name and the column is the fold change after taking the logarithm with the base of 2. [file 3677789.f1.zip › Supplement Figure 1.png]

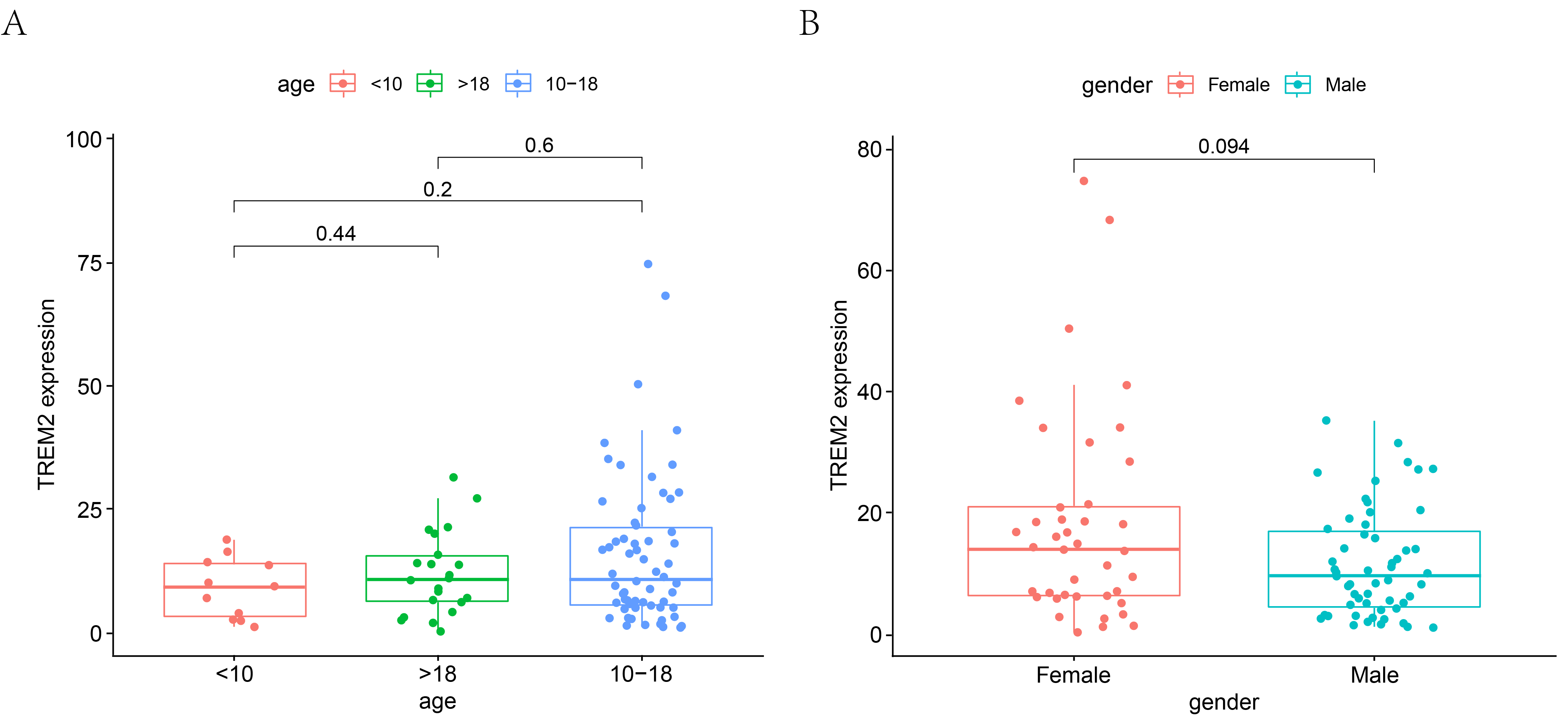

Supplement: Supplementary Materials — The Supplementary Figures and Tables for this article can be found online at: https://doi.org/10.6084/m9.figshare.21972614.v1. Supplementary Figure 1: the GO and KEGG enrichment analysis. (A, B) The GO enrichment analysis. (C, D) The KEGG enrichment analysis. Supplementary Figure 2: relationship between TREM2 expression and clinical features. (A) Relationship between TREM2 expression and age. (B) Relationship between TREM2 expression and gender. Supplementary Table 1: immune infiltration score. The row is the ID of the sample and the column is the score, including StromalScore, ImmuneScore, and ESTIMATEScore. Supplementary Table 2: differentially expressed genes between high and low ImmuneScore groups. The row is the gene name, and the column is the fold change after taking the logarithm with the base of 2 and the adjusted P value in turn. Supplementary Table 3: differentially expressed genes between high and low StromalScore groups. The row is the gene name, and the column is the fold change after taking the logarithm with the base of 2 and the adjusted P value in turn. Supplementary Table 4: common differentially expressed genes shared by ImmuneScore groups and StromalScore groups. The row is the gene name and the column is the fold change after taking the logarithm with the base of 2. [file 3677789.f1.zip › Supplement Figure 2.png]
